# Editorial Notes

**Published:** 1937

**Authors:** 


### Editorial Notes

**The late Mr. James Arrowsmith-Brown.**

When Mr. James Arrowsmith died in 1913 the Editor of the *Journal* wrote of him: "He was always greatly interested in the welfare of the *Journal* . . . we were fortunate in obtaining the assistance of one whose whole heart was in the work."

The good fortune of the *Journal* continued under his nephew and successor, Mr. James Arrowsmith-Brown, whose death we so deeply deplore. There is a popular idea that publishers and writers do not always work in harmony, and that the former live by exploiting the successes of the latter. It is not believed that publishers ever contribute to a writer's success or bear the cost of the failures. However this may be, there is no question of the debt which editors of professional journals owe to their printers and publishers. The editorial committee of a medical journal is especially liable to be a band of amateurs. In our own case we acknowledge to the full the constant help and guidance given to this *Journal* by the late Mr. Arrowsmith-Brown. His judgment was as invaluable as his friendship was precious.

**Tuberculosis Conference in Bristol.**

The Twenty-third Annual Conference of the National Association for the Prevention of Tuberculosis met in Bristol on the 1st, 2nd and 3rd July, in the Physics Laboratory of the University. Owing to ill-health, Sir Robert Philip,

Chairman of the Council of the Association and President of the Conference, was unable to attend. His absence from the Conference, for the first time in twenty-three years, was the more regretted since the session on Saturday, 3rd July, had been specially arranged to commemorate the jubilee of his opening of the first British tuberculosis dispensary at Edinburgh in 1887. The *Medical Officer*, in its editorial comment on the Conference, says that its atmosphere was rather different from that which has ruled in past years. The best thing that can be said about it is that what it did could never have been done before . . .

"In the session devoted to nutrition we heard the phrase 'self-selected diet' running through the discussion as a *leit-motif*. It was used first by Dr. Nixon, Emeritus Professor of Medicine, University of Bristol, in his opening speech, and cropped up with each subsequent speaker, to be used finally in the vote of thanks to the Chairman, Professor Lyle Cummins. It marks a definite advance in our knowledge of nutrition. Before we had a science of nutrition 'eat what you like' was the only guide to our requirements. Now that we know much about the subject it comes back again, but with rather a different meaning. For now we can apply what we know of the needs of man to suit the special needs of each individual. Preventive medicine is becoming the fitting of general physiological science to individual physiological variation. This is the characteristic of 1937 which is permeating all our discussions. Though we do not intend to refer here to the individual papers, we must mention one speaker whose name was not on the schedule and of whose words there is no printed record. We refer to Dr. Fred M. F. Meixner, of Illinois, who attended as the representative of the National Tuberculosis Service of the United States. Dr. Meixner spoke extempore, but he had many important things to say, so we hope that a full report of his speech will be available. What he said clung round the characteristic—the variation of what we know to be generally right to suit the variations of the recipients. He spoke of open-air treatment in Chicago, where for three months of the year the temperature is expected to be below zero, of the swamping of appetite by vast volumes of milk, of the dangers of homework to children who are fatigued by it, and of the influence of maladjustment in the development of adolescent tuberculosis.

"Another note dominating the conference was the dictum—what is right for the management of the diseased is right for the healthy to prevent their becoming diseased. Here caution is required, else we shall get back to 'preventive surgery,' which led us towards disaster. But in general the policy is sound. If ventilation, nutrition and so on are of value in helping the diseased back to health, why not give these advantages earlier and stop them from becoming diseased at all?"

The *Medical Officer* mentions another less encouraging feature of this Conference: "His Majesty's Government debarred school medical officers from taking part in a discussion on open-air schools."

Now what exactly is His Majesty's Government afraid of? A sort of climatic independence that is bred in Bristol?

**Elections to United Honorary Staffs of B.R.I. and B.G.H.**

THE first elections to the united honorary medical staffs of the Royal Infirmary and General Hospital were made on 25th May, 1937, when the election committees of the two institutions met in the Board Room of the Royal Infirmary as a Single Committee. This step was adopted to bring into existence a single Radiological Department. Drs. F. G. Bergin, G. B. Bush and S. B. Adams were appointed to the charge of the new department. In the case of the two former the elections confirmed them in duties they had formerly discharged to the General Hospital only. Dr. Adams is a graduate of Bristol University, whom we welcome back as the "first foot" on the "amalgamation" threshold.

